# Spheroids of Endothelial Cells and Vascular Smooth Muscle Cells Promote Cell Migration in Hyaluronic Acid and Fibrinogen Composite Hydrogels

**DOI:** 10.34133/2020/8970480

**Published:** 2020-02-19

**Authors:** Xingang Zuo, Haolan Zhang, Tong Zhou, Yiyuan Duan, Hao Shou, Shan Yu, Changyou Gao

**Affiliations:** ^1^MOE Key Laboratory of Macromolecular Synthesis and Functionalization, Department of Polymer Science and Engineering, Zhejiang University, Hangzhou 310027, China; ^2^Dr. Li Dak Sum & Yip Yio Chin Center for Stem Cell and Regenerative Medicine, Zhejiang University, Hangzhou 310058, China

## Abstract

Cell migration plays a pivotal role in many pathological and physiological processes. So far, most of the studies have been focused on 2-dimensional cell adhesion and migration. Herein, the migration behaviors of cell spheroids in 3D hydrogels obtained by polymerization of methacrylated hyaluronic acid (HA-MA) and fibrinogen (Fg) with different ratios were studied. The Fg could be released to the medium gradually along with time prolongation, achieving the dynamic change of hydrogel structures and properties. Three types of cell spheroids, i.e., endothelial cell (EC), smooth muscle cell (SMC), and EC-SMC spheroids, were prepared with 10,000 cells in each, whose diameters were about 343, 108, and 224 *μ*m, respectively. The composite hydrogels with an intermediate ratio of Fg allowed the fastest 3D migration of cell spheroids. The ECs-SMCs migrated longest up to 3200 *μ*m at day 14, whereas the SMC spheroids migrated slowest with a distance of only ~400 *μ*m at the same period of time. The addition of free RGD or anti-CD44 could significantly reduce the migration distance, revealing that the cell-substrate interactions take the major roles and the migration is mesenchymal dependent. Moreover, addition of anti-N-cadherin and MMP inhibitors also slowed down the migration rate, demonstrating that the degradation of hydrogels and cell-cell interactions are also largely involved in the cell migration. RT-PCR measurement showed that expression of genes related to cell adhesion and antiapoptosis, and angiogenesis was all upregulated in the EC-SMC spheroids than single EC or SMC spheroids, suggesting that the use of composite cell spheroids is more promising to promote cell-substrate interactions and maintenance of cell functions.

## 1. Introduction

Cell migration plays an important role in many biological processes [1] ranging from wound healing [2], tissue development [3], revascularization [4], immune response [5], etc. For example, endothelial cells and smooth muscle cells participate in the process of formation of blood vessels, where appropriate migration of the desired types of cells is preferable. The process of tumor metastasis also involves the migration of cancer cells [6]. However, so far, the study of cell migration has mainly focused on planar substrates which are convenient for the direct observation by live microscopy and measurement of cell-substrate interactions such as cell adhesion force. The cell mobility *in vitro* is influenced by the gradient distribution of ligands or signaling molecules [7, 8], surface topology [9], and material modulus [10]. For example, the migration rate of smooth muscle cells is mediated by the gradient distribution of VAPG on a surface [7]. Although the principles obtained are basically applicable to 3-dimensional cell-biomaterial interactions, they may not be able to match exactly the case *in vivo*, where cells usually interact with their extracellular matrices (ECMs) in 3 dimensions. The ECM is known to regulate many cell behaviors such as cell adhesion and migration by affecting molecular interaction and signal transduction [11, 12].

Besides the cell-substrate interactions, the cell-cell interactions are also pivotal in mediating many cellular behaviors including cell migration [13, 14]. The collective cell migration in a cell sheeting model can disclose characteristics of cell-cell interactions more comprehensively [15–17]. For example, Friedl and Gilmour studied the characteristics of collective cell migration in tumor [13], and Qin et al. used a light-sensitive hydrogel to induce the collective cell migration to a specific direction [18]. In general, the collective cell migration can better mimic the true cell migration environment *in vivo* and provide the basis for better design of biomaterials. Besides, the intercellular communications take place not only among the same kind of cells but also the different types of cells *in vivo*. Hsu et al. found that human mesenchymal stem cells and endothelial progenitor cells cospheroids have a greater angiogenic effect *in vitro* [19]. Korff et al. explored the effect of coculture cell spheroids of endothelial cells and smooth muscle cells on angiogenesis [20].

Nonetheless, these pioneering studies have focused mainly on the angiogenesis of cell spheroids in hydrogels, whereas the cell-cell and cell-substrate interactions that govern the collective cell migration have not been considered simultaneously. Therefore, integration of the cell spheroids with an appropriate material system would be a suitable model to explore the fundamental cell-cell and cell-substrate interactions and the behaviors of collective cell migration. Among the various biomaterials with 3D structures, the hydrogels stand for an ideal model to study the 3D cell-matrix interaction and migration because of their similar physiochemical structures and properties to natural ECM [21, 22] and their definite 3-dimensional entrapment of cells for tissue engineering and regenerative medicine [23, 24]. So far, various types of hydrogels with adjustable modulus, controllable degradation, and designable chemical compositions have been designed to simulate the microenvironment *in vivo*. Considering that the environment *in vivo* is not static and contains many different gradients, stimulus-responsive hydrogels triggered by light irradiation [18], pH change [25], and enzyme catalyzation [26] have been developed as well.

**Scheme 1:**
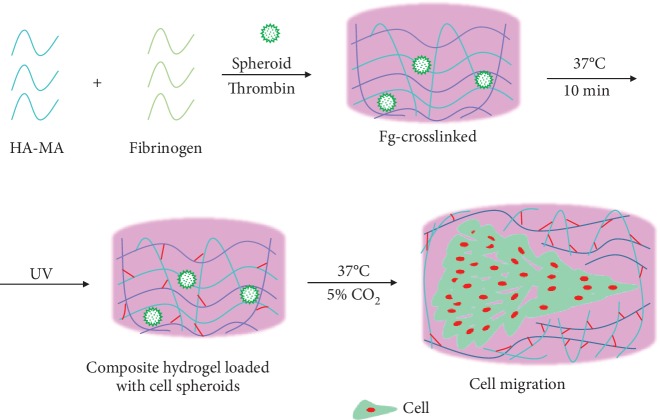
Scheme of fabricating MA-HA and fibrinogen composite hydrogel, in which EC, SMC, and EC-SMC spheroids are encapsulated and the migration of cells from the cell spheroids into the hydrogels is measured and compared. The fibrinogen is crosslinked by thrombin, and MA-HA molecules having unsaturated carbon double bonds are subsequently crosslinked by UV irradiation under the existence of photoinitiator I2959.

Although the collective cell migration has been analyzed previously, most of the previous studies are performed on 2D planar substrates with a focus on cell-cell interactions by using a single type of cells. In this work, a pioneering model is designed to study the collective cell migration behaviors of (cocultured) cell spheroids in composite and dynamic 3D hydrogels, by taking into account simultaneously the influence of cell-cell and cell-substrate interactions (Scheme 1). From the viewpoint of biomaterial science, these two types of interactions are the core scientific questions governing the biological performance of biomaterials in regenerative medicine. In particular, the dynamic hydrogel matrix rather than a standard culture dish or traditional biomaterials can better mimic the ECM *in vivo*, signifying the importance and intrinsic connection of the obtained results with tissue engineering and regenerative medicine. Some important phenomena are indeed observed for the first time, whose mechanisms are further explored. The fundamental findings are of significance in disclosing the intricate relationship between cells and cell-substrate by better mimicking the dynamic matrix and cell-cell interactions in spheroids, which is the case *in vivo*.

Specifically, the 3D migration behaviors of 3 types of cell spheroids, i.e., endothelial cell (EC), smooth muscle cell (SMC), and EC-SMC composite spheroids, were investigated in hyaluronic acid (HA)-fibrinogen (Fg) composite hydrogels (Scheme 1). We chose HA and Fg to prepare the dynamic hydrogels because they are both biologically originated and degradable with good bioactivity to cells. HA and its derivatives have been fabricated into hydrogels for different applications in regenerative medicine [27, 28]. Fg is one of the major proteins in plasma and possesses some cell-signaling domains [29, 30]. It is more easily degraded and released to provide necessary space for cell migration in the HA-Fg hydrogels. In our system, Fg is gelated by thrombin to form the first crosslinking networks, and then the methacrylated HA is crosslinked under UV initiation to form the composite hydrogels, in which cell spheroids are loaded. Both ECs and SMCs are mandatory for the angiogenesis and maturation of new blood vessels [31–33]. The migration of encapsulated cell spheroids was then monitored and quantified by laser scanning microscopy after being cultured for different periods of time.

## 2. Results

### 2.1. Characterization of Composite Hydrogels

The hydrogels based on hyaluronic acid are applied widely as a matrix for tissue regeneration due to their similarity in chemical and physical structures to natural extracellular matrix. In this work, the HA-MA was synthesized by transesterification between HA with methacrylic anhydride (MA) ([Supplementary-material supplementary-material-1]). ^1^H NMR spectra ([Supplementary-material supplementary-material-1]) show that the integrated peak areas of carbon double bonds (5.6 and 6.1 ppm) and –CH_3_ (1.84 and 1.75 ppm for the methyl groups of both the HA backbone and the coupled methacrylate) were 0.445 and 1, respectively, according to which the substitution degree of –OH groups in HA was 50%, namely, 2 –OH groups were methacrylated in each repeating unit of HA-MA macromolecules [34–36].

**Figure 1:**
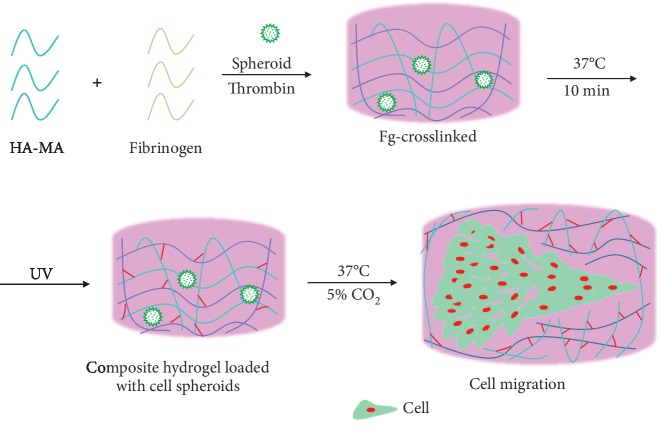
(a) CLSM images showing the distribution of FITC-fibrinogen in composite hydrogels. (1, 4), (2, 5), and (3, 6) represent the HA-MA/Fg(1/3), HA-MA/Fg(1/1), and HA-MA/Fg(3/1) hydrogels, respectively. (1-3) The typical cross section and (4-6) 3D reconstructed images. (b) Swelling ratio, (c) compression modulus, and (d) weight loss in water (after lyophilization) of different hydrogels. (e) Fibrinogen released from the composite hydrogels in PBS. ∗ indicates significant difference at *p* < 0.05 level.

HA-MA was then mixed with different ratios of fibrinogen to prepare the composite hydrogels.  Figure 1(a) shows that the FITC-tagged fibrin networks distributed evenly in the composite hydrogels. The fluorescence intensity of hydrogels decreased along with the increase of HA-MA ratio, which is consistent with the decreased content of Fg.

The swelling ratio of the hydrogels was enlarged along with the increase of HA-MA ratio (Figure 1(b)), revealing the more hydrophilic nature of HA molecules compared with Fg.  Figure 1(c) shows that the compressive modulus was doubled when the HA-MA/Fg (including Fg and thrombin) volume ratio increased from 1/3 to 1/1, but was not further improved when the ratio was enlarged to 3/1. This result reveals the natural difference between the HA-MA and Fg networks again, and the HA-MA may result in hydrogels with a higher crosslinking degree and thereby larger mechanical strength compared with Fg [37]. This conclusion is partially substantiated by the microstructure of lyophilized hydrogels ([Supplementary-material supplementary-material-1]), where fragile pieces and integrate porous structures were observed for the HA-MA/Fg(1/3) and HA-MA/Fg(3/1) hydrogels, respectively.

All the composite hydrogels lost their weight rapidly after being incubated in water for only 1 day, which did not change significantly over an incubation period of 14 days (Figure 1(d)). At 14 days, the weight loss for HA-MA/Fg(1/3), HA-MA/Fg(1/1), and HA-MA/Fg(3/1) was about 65%, 50%, and 35%, respectively. This weight loss led to the decrease of compressive modulus to almost half of their original values, i.e., from about 8 kPa to 4 kPa for the HA-MA/Fg(1/1) and HA-MA/Fg(3/1) hydrogels, and from about 4 kPa to 2 kPa for the HA-MA/Fg(1/3) hydrogel, respectively ([Supplementary-material supplementary-material-1]).

To verify the structure variation during this process, the hydrogels before and after degradation were subjected to FTIR characterization ([Supplementary-material supplementary-material-1]). There were two typical peaks that can identify the structure change: 1660 cm^−1^ for C=O of HA-MA and fibrinogen, and 1167 cm^−1^ for unreacted –C=C–H of HA-MA. The intensity ratio of C=C-H/C=O increased after degradation for all the three composite hydrogels ([Supplementary-material supplementary-material-1]), suggesting that the component of Fg was reduced in the degraded hydrogels. Hence, the significant weight loss of the hydrogels is most possibly attributed to the loss of Fg component. Indeed, although the absolute weight loss of the hydrogel was smaller than the theoretical values of Fg in the composite hydrogels (85%, 65%, and 38% for HA-MA/Fg(1/3), HA-MA/Fg(1/1), and HA-MA/Fg(3/1), respectively), it was very close to the measured values of Fg in the hydrogels (75.1%, 49.0%, and 33.5% for HA-MA/Fg(1/3), HA-MA/Fg(1/1), and HA-MA/Fg(3/1), respectively. These values were obtained by subtracting the lyophilized weight of HA-MA hydrogels from the HA-MA/Fg hydrogels prepared at the same conditions.

Although the fibrin hydrogel is usually degraded faster than the HA hydrogel due to its physical crosslinking nature [38, 39], it is too fast to degrade the Fg completely within only 1 day. Indeed, we found that the pure Fg hydrogels became powder completely after lyophilization, which was fully soluble in water.  Figure 1(e) shows that only very small amount of fibrinogen was released from the hydrogels (without lyophilization) within 1 day. Along with time prolongation, the Fg was released almost linearly, with the highest amount for the HA-MA/Fg(1/3) and lowest amount for the HA-MA/Fg(3/1), respectively. These results demonstrate that Fg was released in a sustainable way from the hydrogels rather than the drastic change as shown in Figure 1(d).

### 2.2. Characterization of Cell Spheroids

**Figure 2:**
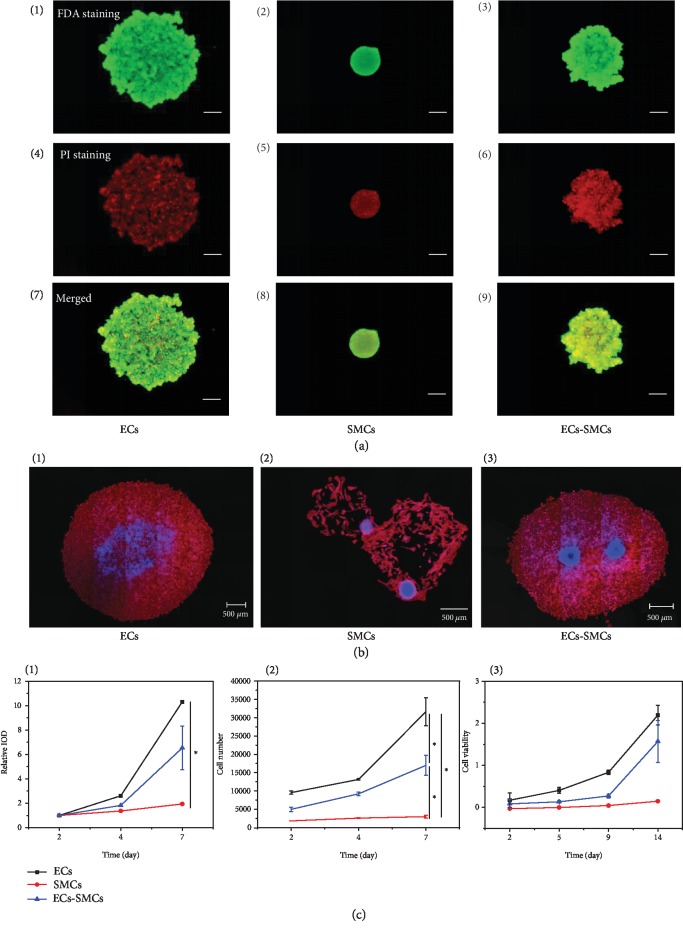
(a) Fluorescent images of (1, 4, 7) EC, (2, 5, 8) SMC, and (3, 6, 9) EC-SMC spheroids after being stained by (1-3) FDA and (4-6) PI, respectively. (7-9) Corresponding merged images. These cell spheroids were just transferred into normal culture plate. Green and red represent live and dead cells, respectively. Scale bar: 200 *μ*m. (b) CLSM images of (1) EC, (2) SMC, and (3) EC-SMC spheroids after being cultured for 3 days on TCPS, respectively. The cytoskeleton and cell nuclei were stained with rhodamine-labeled phalloidin (red) and DAPI (green), respectively. (c) Cytoviability and proliferation assay of the cell spheroids. (1) MTT assay and (2) cell number after the cell spheroids were cultured on TCPS for different times. (3) Viability of EC, SMC, and EC-SMC spheroids after they were cultured on the surface of HA-MA/Fg(1/1) composite hydrogel for different times.

In most previous studies, the attention has been paid to the cell-substrate interactions in terms of cell migration, revealing that the cell adhesion force is the intrinsic factor governing cell migration rate [40, 41]. Collective cell migration has also been studied by culturing cell sheet on 2D substrate, which can simultaneously reveal the cell-substrate and cell-cell interactions [42]. In most real situations *in vivo*, however, the cells interact with surrounding environment and other cells in a 3D manner. Therefore, cell aggregates such as the cell spheroids would be an ideal model to study the collective cell migration behavior 3 dimensionally. In this study, 3 types of cell spheroids were prepared by using the same number of cells and were observed by fluorescent microscopy after FDA (green, live cells) and PI (red, dead cells) staining (Figure 2(a)). The cells were combined loosely with each other in the EC spheroids, whereas the cells were densely accumulated with each other in the SMC spheroids, and the cells in the composite EC-SMC spheroids showed the intermediate packing state. These accumulate states were consistent with their average diameters, which were 343, 108, and 224 *μ*m for the EC, SMC, and EC-SMC spheroids, respectively. Most of the cells, in particular those in the outer region of the spheroids, were viable (Figure 2(a)1-3, 7-9), whereas there were some dead cells in the inner region (Figure 2(a)4-6) due to the known reasons of limitation of nutrition exchange and hypoxia environment [43]. After these cell spheroids were cultured on TCPS for 3 days, all the cells could migrate from the spheroids and adhere on the culture plate, forming well spreading morphology (Figure 2(b)). The blue color in the spreading spheroids represents the original positions of the cell spheroids. Optical microscopy observed similar migration of cells from the spheroids ([Supplementary-material supplementary-material-1]), where the EC spheroids were not noticeable due to the very well spreading of ECs. Moreover, the EC-SMC composite spheroids were further observed by fluorescence microscopy after they were stained with Cell Tracker™ Green CMFDA (green) and Cell Tracker™ Orange CMTMR (red) in prior of spheroid formation, respectively. After coculture for 4 days ([Supplementary-material supplementary-material-1]), both types of cells could migrate from the spheroids, forming composite cell layers nearby the spheroids ([Supplementary-material supplementary-material-1]). However, only ECs were observed far away from the spheroids ([Supplementary-material supplementary-material-1]), revealing that ECs would lead the migration outward. In this region, some red dots existed in the EC. The red dots were most possibly the exosomes secreted by SMCs [44, 45] and were internalized by the ECs. This phenomenon suggests the existence of interactions between ECs and SMCs, which may bring influences on their mobility and functions [46].

Since some dead cells existed in the spheroids, the proliferation of cells in the spheroids was characterized by both MTT assay (Figure 2(c)1) and DNA content assay (Figure 2(c)2). Along with time prolongation, the numbers and viability of cells in the EC and EC-SMC spheroids increased monotonously, whereas those of cells in the SMC spheroids did not change obviously. Comparatively, the ECs proliferated fastest with a significantly high cell number and viability at day 14. Furthermore, the cell spheroids were also cultured inside HA-MA/Fg(1/1) composite hydrogels, and their viability was determined by CCK8 assay (Figure 2(c)3). Different from the culture on TCPS, the viability of cells in all the spheroids increased along with time prolongation, with the lowest for the SMC spheroids at all culture time points. Again, the EC spheroids had the largest viability, but eventually had the same value with EC-SMC spheroids at day 14. These results reveal that the cells in the spheroids maintain their ability to proliferate, particularly inside the composite hydrogel.

### 2.3. 3D Cell Migration in Hydrogels

**Figure 3:**
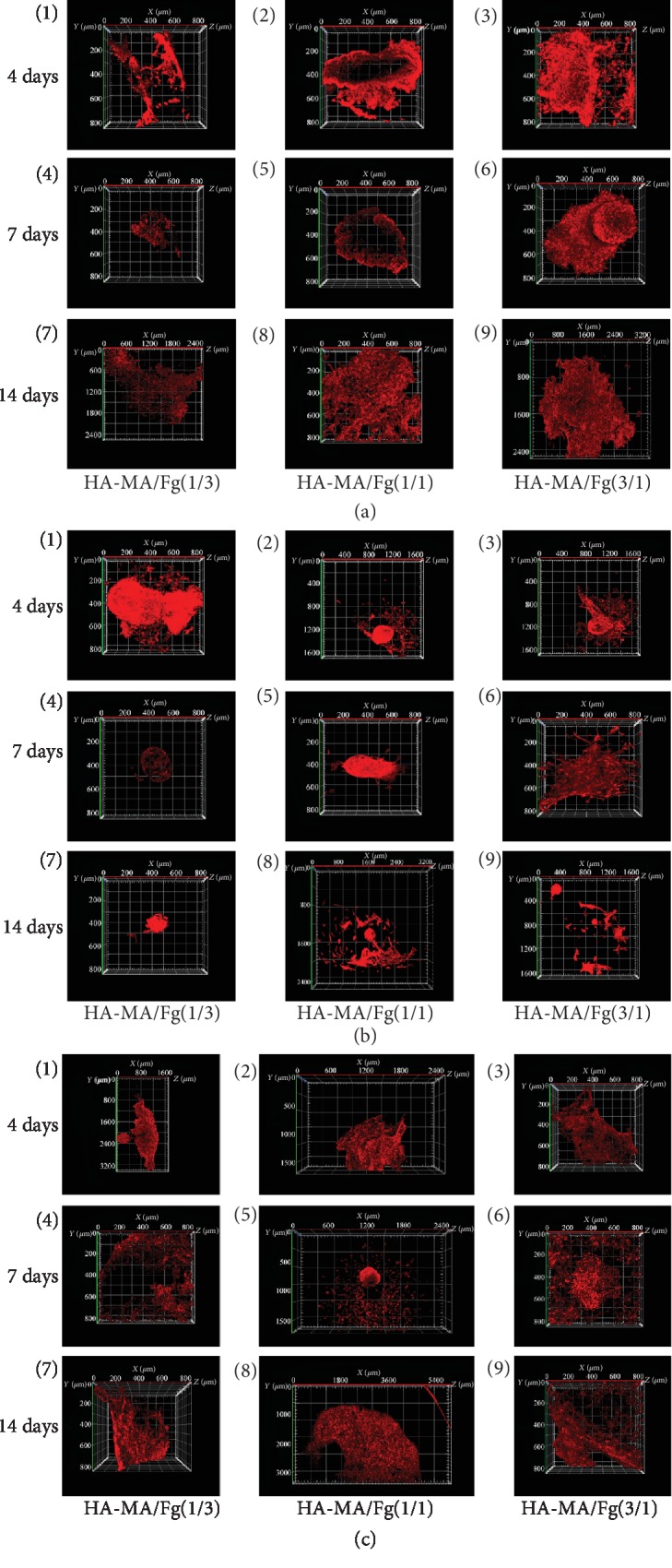
CLSM images of (a) EC, (b) SMC, and (c) EC-SMC spheroids encapsulated in (1, 4, 7) HA-MA/Fg(1/3), (2, 5, 8) HA-MA/Fg(1/1), and (3, 6, 9) HA-MA/Fg(3/1) composite hydrogels for (1-3) 4, (4-6) 7, and (7-9) 14 days, respectively. To show the whole contours of the cells, the scale bars are different and can be referred to each image.

Next, the cell spheroids were mixed with the three types of composite hydrogel precursors, which were then formed hydrogels at mild conditions. The *in situ* entrapped cell spheroids were cultured *in vitro*, and their 3D migration behaviors were investigated by CLSM over a period of 14 days. Representative images for EC, SMC, and EC-SMC spheroids at 4, 7, and 14 days are shown in Figures 3(a)–3(c) after the cytoskeleton and cell nuclei were stained with rhodamine-labeled phalloidin (red) and DAPI (green), respectively. It has to mention that the hydrogels were severely stained by DAPI as well, and hence, the nuclei were not shown in these images. Generally, the cell spheroids in the hydrogels migrated to different directions, and the cell invasion area increased over time.

**Figure 4:**
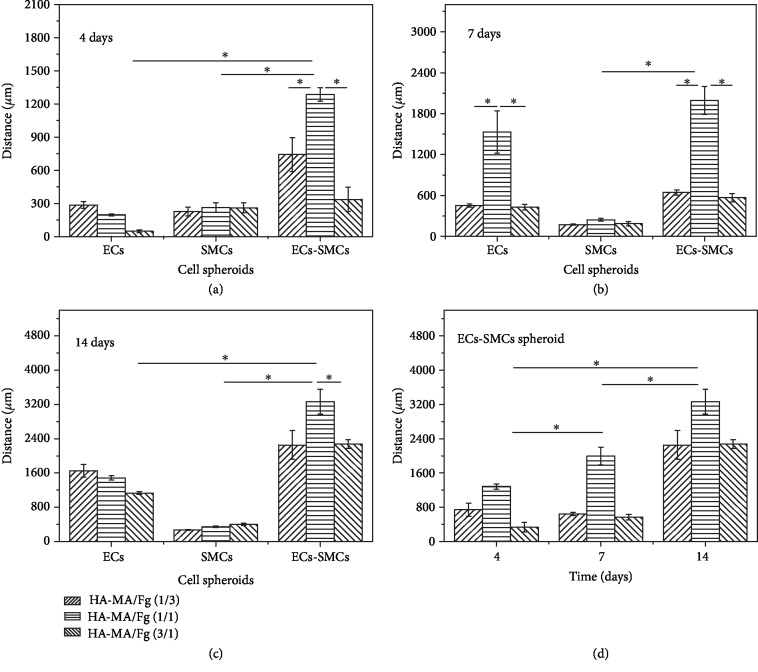
Migration distance of EC, SMC, and EC-SMC spheroids in different hydrogels after being cultured for (a) 4, (b) 7, and (c) 14 days. The migration distance is defined as the entire diameter of the cell contour minuses the diameter of the cell spheroid. (d) For the convenient and direct comparison, the migration distance of EC-SMC spheroids cultured in different hydrogels was replotted vs. culture time. ∗ indicates significant difference at *p* < 0.05 level.

Quantitative analysis of the cell migration distance is shown in Figure 4. Overall, the migration distance of all the cell spheroids increased along with time prolongation in all types of hydrogels, but their extents were different. At each time point, the EC-SMC spheroid showed the significantly longer migration distance in the HA-MA/Fg(1/1) hydrogel with a value of 3200 *μ*m at day 14. The EC spheroids migrated also faster compared with the SMC spheroids, which had the slowest mobility with a final migration distance of only ~400 *μ*m at day 14. It is worth to mention that the SMC spheroids migrated faster during the first 4 days and then slower during the next 10 days ([Supplementary-material supplementary-material-1]). To better reveal the phenomenon, the migration distance of the EC-SMC spheroids *vs*. time was replotted in one figure (Figure 4(d)), substantiating the conclusion of monotonous increase of migration distance over time and the strongest mobility in the HA-MA/Fg(1/1) hydrogels.

These results confirm that both the physicochemical properties of hydrogels and the types of cell spheroids strongly influence the cell migration behaviors in 3D matrices. The HA-MA/Fg(1/1) hydrogels turned out to be the most suitable one to allow fastest cell migration, in particular for the EC-SMC spheroids. It is known that the compressive modulus and swelling ratio may affect the cell migration behaviors [47, 48], and cocultured cells could promote the cell migration rate on a two-dimensional material surface [19], revealing that the communications among cells are vital for regulating cell mobility.

As a comparison, the migration distance of different cell spheroids was also measured on TCPS ([Supplementary-material supplementary-material-1]). Although the migration distance of EC, SMC, and EC-SMC spheroids increased along with the prolongation of culture time, the exact patterns were different from those in 3D hydrogels. The shortest distance was still found for the SMC spheroids. However, the longest distance was achieved by the EC spheroids. Moreover, the absolute migration distance at day 14 was 8, 10, and 4 folds higher for the same EC, SMC, and EC-SMC spheroids migrating in the HA-MA/Fg(1/1) hydrogels. These results reveal the significant difference of cell migration in 3D hydrogels and on 2D planar substrate.

In our study, the fibrinogen crosslinked by thrombin in the composite hydrogels was released with a faster rate, which may create space for cells to invade into the hydrogels. Moreover, fibrinogen, a type of plasma proteins, can interact with cells to offer necessary anchoring sites for cells [49]. In contrast, due to its super hydrophilicity, the HA molecule usually shows a rather strong cell-resisting property [50]. However, there also exist CD44 ligands in the HA molecule, which may interact with the specific receptors on membranes of some types of cells such as ECs and SMCs [51, 52]. These cell-substrate interactions are mandatory when the cells adopt the mesenchymal migration mechanism [53].

**Figure 5:**
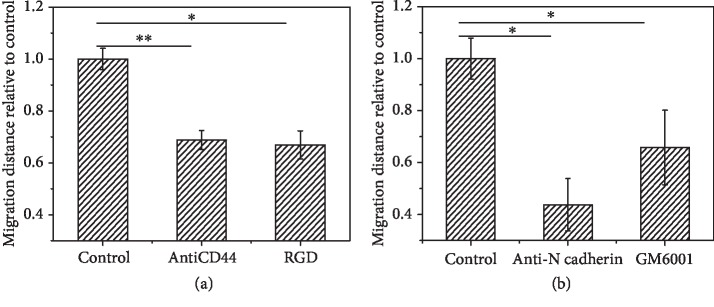
Relative migration distance of EC-SMC spheroids encapsulated in HA-MA/Fg(1/1) hydrogels for 7 days (a) without or with 20 *μ*g/mL CD44 antibody or 40 *μ*g/mL free RGD in culture medium and (b) without or with 10 *μ*g/mL anti-N-cadherin antibody or 10 *μ*g/mL GM6001 in culture medium, respectively. ∗ indicates significant difference at *p* < 0.05 level.

Taking the HA-MA/Fg(1/1) hydrogels as a typical example, the migration of EC-SMC spheroids was studied by adding 20 *μ*g/mL CD44 antibodies or 40 *μ*g/mL free RGD in the culture medium, which can block the cell-substrate interactions [54]. Again, the cell migration was monitored by CLSM after being cultured for 7 days ([Supplementary-material supplementary-material-1]. The cytoskeleton was stained with rhodamine-labeled phalloidin).  Figure 5(a) shows that the migration ability of the cell spheroids was significantly reduced, and the net distance was only 2/3 of the blank control in both cases. Therefore, the cell-substrate interaction surely takes an important role in governing the migration of EC-SMC spheroids.

Different from the case of single-cell migration, the cell-cell interactions must come into play in the 3D cell migration [55]. To explore this influence, the culture medium was supplemented with 10 *μ*g/mL anti-N-cadherin antibody, which is known to inhibit cell-cell interactions because the N-cadherin is one of the major proteins binding cells [56]. In this case, the migration distance in the HA-MA/Fg(1/1) hydrogels ([Supplementary-material supplementary-material-1]) was reduced to 50% of that in the blank control (Figure 5(b)), demonstrating that the cell-cell interactions also intrigue the migration of cell spheroids. These results substantiate that the collective cell migration is easier to take place in comparison with the single-cell migration (random migration) in hydrogels [57].

Moreover, degradation of the matrices by cells is important to create necessary space for cell invasion. The matrix metalloproteinase (MMP) secreted by cells is known to degrade matrices during cell culture. Hence, the culture medium was supplemented with 10 *μ*g/mL GM6001 (a type of inhibitors for MMP enzyme). In this case, the net migration distance was also reduced significantly, with a value of 2/3 of the blank control (Figure 5(b)).

Taking all these results into consideration, the degradation of hydrogels by MMP secreted by cells, and the cell-substrate and cell-cell interactions all come into play for governing the 3D cell migration in the HA-Fg composite hydrogels. It is likely that the HA-MA/Fg(1/1) hydrogels happen to be the most suitable one to allow the fastest migration of cell spheroids, in particular the EC-SMC spheroids.

### 2.4. Expression of Genes

**Figure 6:**
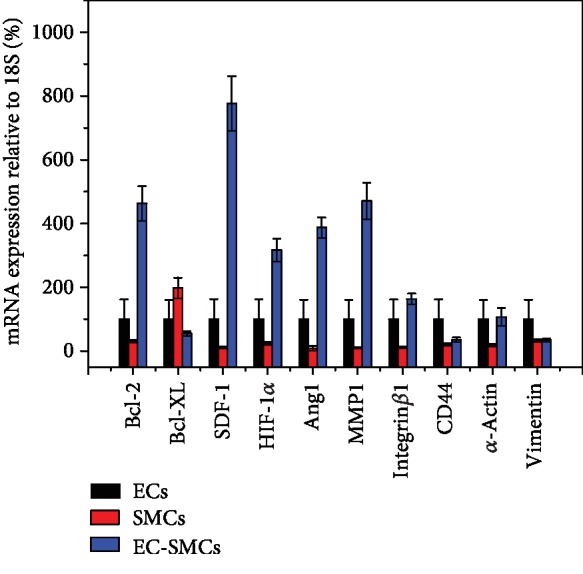
Quantitative real-time polymerase chain reaction (qRT-PCR) analysis of genes related to cell apoptosis, vascularization, and migration expressed by EC, SMC, and EC-SMC spheroids in HA-MA/Fg(1/1) hydrogels.

To gain deeper insight into the influences of different types of cell spheroids on cell migration in the HA-MA/Fg(1/1) hydrogels, the expression of important genes related with cell adhesion (integrin *β*1, CD44, *α*-actin, MMP-1, and vimentin), apoptosis (Bcl-2 and Bcl-XL), and angiogenesis (SDF-1, HIF-1, and angiopoietin 1) was analyzed by using RT-PCR. The expression of MMP was also compared.  Figure 6 shows that most of these genes were highly expressed in the EC-SMC spheroids followed with the EC spheroids, compared with those in the SMC spheroids being cultured similarly in the HA-MA/Fg(1/1) hydrogels. The MMP-1 and integrin *β*1 were expressed with the highest values in the EC-SMC spheroids, whereas the expression of CD44, *α*-actin, and vimentin had no significant difference among the different cell spheroids. These results confirm again that the degradation of matrices, and interactions of cells and matrices are both important for the migration of cells in the hydrogels.

It is known that the relatively oxygen-deficient environment in cell spheroids can better help the cells adapt in the three-dimensional materials [43]. Although the expression of Bcl-XL was not different among the three types of cell spheroids, the expression level of Bcl-2 in the EC-SMC spheroids was significantly improved. This would mean that the cells in this composite spheroid can better maintain their viability against death in the HA-MA/Fg(1/1) hydrogels, leading to stronger mobility.

On the other hand, the culture of EC-SMC composite spheroids in the hydrogels can stimulate angiogenesis in 3D environment *in vitro* to some extent.  Figure 6 shows that the expression of SDF-1, HIF-1, and angiopoietin 1 was all significantly improved in the EC-SMC spheroids compared with that in the EC or SMC alone spheroids, suggesting that the coculture cell spheroid is more conducive for angiogenesis.

## 3. Discussion

The collective cell migration in a matrix is important for many biological processes such as wound healing, tissue regeneration, immune response, and metastasis [43, 58]. It involves both cell-substrate and cell-cell interactions, which govern the cell migration behaviors [59, 60]. The migration of cells into (not toward) 3D materials needs to push aside or degrade the environmental matrix, which requires much more energy input than that on 2D planar substrate [61]. In this regard, the cell-cell interaction is much more important, and thus, the collective cell migration may be easier compared with the single-cell migration (random migration). In this study, three types of cell spheroids, i.e., EC, SMC, and EC-SMC spheroids, were prepared, and their 3D migration behaviors were monitored and quantified in the HA-Fg composite hydrogels. The Fg in the composite hydrogels was released gradually, which might then offer necessary space for cell migration. The results show that the HA-MA/Fg(1/1) hydrogels with an intermediate content of Fg allowed the fastest migration of cell spheroids. The EC-SMC spheroids had the strongest mobility in the composite hydrogels and reached the longest migration distance in the HA-MA/Fg(1/1) hydrogels. In contrast, the SMC spheroids showed the slowest migration rate. In this system, both the 3D cell-substrate and cell-cell interactions take pivotal roles, which are further confirmed by expression of genes related with cell adhesion. Moreover, RT-PCR results reveal that the EC-SMC spheroids in the HA-MA/Fg(1/1) hydrogels could better maintain their viability against apoptosis, so there are more living cells for migration. Moreover, the coculture cell spheroids also show the potential to promote angiogenesis compared with the ECs or SMC spheroids alone.

Many types of biomaterials [62–64] with different physicochemical properties can influence on cell migration on the 2D planar substrates [47, 65]. However, three-dimensional materials are more capable of simulating the real cell niche *in vivo*, allowing the simultaneous interactions between cell-cell and cell-matrix. The interactions among cells affect the functions of cells in cell spheroids. For example, endothelial cell spheroids are more likely to participate in the formation of blood vessels, and bone marrow mesenchymal stem cell spheroids are more prone to chondrogenic differentiation [20, 66]. However, none of the previous studies has considered the effects of materials on 3D cell migration throughout the processes as well as clarification of the mechanisms behind. Although the 3D biomaterials have been extensively studied in terms of influences on cell morphology, polarization, and differentiation, little attention has been paid to their effect on cell migration. Trappmann et al. studied the behaviors of cells in 3D tubular materials with different degradation properties to explore the conditions for the formation of vascularization [67]. In addition, the collective cell migration is more beneficial to the study of cell-cell interactions compared to the traditional single-cell migration [55]. Herein, we found that the EC-SMC spheroids had the fastest migration rate in the HA-MA/Fg(1/1) hydrogels, revealing that the interactions between EC and SMC promote the cell migration. Furthermore, it is likely that the appropriate ratio and release rate of Fg from the HA-MA/Fg(1/1) hydrogels benefit the fast migration as well.

Previous studies have shown that multicellular aggregates have their unique properties and are more capable of mimicking cell migration *in vivo* compared to single cells [58, 68]. The cell spheroid model creates a hypoxic environment and allows cells to adapt to it. The 3D microenvironment is hypoxic or less nutrient since the medium and oxygen are hardly to exchange quickly. Single cells in this environment are difficult to maintain the original characteristics or even survive. However, previous studies have shown that stem cell spheroids are more conducive to maintain stemness in 3D environment [69]. In our study, we focused on the collective cell migration behaviors of (cocultured) cell spheroids in composite and dynamic 3D hydrogels (Figure 1), by taking into account simultaneously the influence of cell-cell and cell-substrate interactions. We indeed found some important phenomena that have never been observed before, and clarified and suggested the mechanisms behind. For example, the cocultured cell spheroids allowed the sufficient interactions between different types of cells, leading to the fastest migration rate and secretion of larger amounts of cytokines representing the angiogenesis in the HA-MA/Fg(1/1) composite hydrogels (Figures 4 and 6). Moreover, compared with that of the EC-SMC spheroids, the proliferation rate of EC spheroids was similar on the hydrogel surface, but was higher on TCPS (Figure 2(c) and [Supplementary-material supplementary-material-1]). However, the migration rate in 3D hydrogels was opposite. Therefore, cell proliferation should have no or less relevance to the 3D migration of cell spheroids in this study.

There must be large enough space for cells to migrate in materials [70]. The mesh size of hydrogels depends on the distance between the macromolecular chain grids [71], which is usually a few to tens nanometers. Hence, cells must degrade the surrounding materials to obtain large enough space for migration. During this process, cells also need to interact with the matrices to enable strong enough force for adhesion and migration. The HA-MA/Fg(1/3) hydrogels had the highest content of fibrinogen, leading to fastest Fg release and thereby largest space. However, the migration of cell spheroids was slow in this hydrogel. It is likely that this hydrogel may gradually collapse after Fg release due to its critically high Fg content and lowest modulus (Figure 1c), leading to partial collapse of its 3D structure. In contrast, the HA-MA/Fg(1/1) hydrogel had large enough Fg content and modulus and thus was the most appropriate one to allow fastest migration of cell spheroids.

There are two major mechanisms involving in 3D cell migration: mesenchymal migration and amoeboid migration [72]. The anchorage-dependent cells such as ECs and SMCs need to adhere on substrate and then migrate following the mesenchymal mechanism, which is the case as demonstrated in Figure 5(a). This is consistent with previous finding that cell migration in hydrogels adopts a mesenchymal mechanism as a result of focal adhesion formation [70]. Lei et al. found that if the density of free RGD peptides in hydrogels is higher, the mouse mesenchymal stem cells almost cannot migrate, although the cells can spread faster in the same hydrogels [73]. Besides, Fg/HA can also interact with cells by CD44 ligand likely via the Rho pathway [74, 75]. Moreover, cells can secrete hyaluronidase and MMPs to degrade the composite hydrogels during the cell invasion and migration.

In summary, there are many factors influencing the 3D migration of cell spheroids in the hydrogels, and the interactions between cell-substrate and cell-cell take the major role. The degradation of hydrogels by cell-secreted enzymes or/and release of one component is also important to offer large enough space. These factors may further interplay with each other, leading to a more complicated environment for the cell spheroids to migrate. The cell spheroid model not only mimics the collective migration behaviors of cells *in vivo* but also benefits the survival of cells in hydrogels. The composite cell spheroids can show more biological functions than a single kind of cell spheroids due to the existence of communications between different types of cells. Moreover, the structure of the hydrogels can be further optimized to endow with smarter adaptability to the entrapped cells, offering a more appropriate 3D environment for cell migration and thereby tissue regeneration.

## 4. Conclusions

The composite hydrogels comprising of different ratios of HA-MA and fibrinogen were fabricated. The Fg was uniformly distributed in the hydrogels and could be released gradually along with the prolongation of time. Three types of cell spheroids, i.e., EC, SMC, and EC-SMC spheroids, were prepared by using the same number of cells, showing the largest and smallest in diameter for the ECs and SMCs, respectively. Their 3D migration behaviors were monitored and quantified in the composite hydrogels, revealing that the HA-MA/Fg(1/1) hydrogels with an intermediate content of Fg allowed the fastest migration of cell spheroids. The EC-SMC spheroids had the strongest mobility in the composite hydrogels and reached the longest migration distance in the HA-MA/Fg(1/1) hydrogels. The SMC spheroids were densely packed and migrated slowest in the hydrogels. It was demonstrated that both the cell-substrate and cell-cell interactions took pivotal roles in cell migration. Moreover, the EC-SMC spheroids in the HA-MA/Fg(1/1) hydrogels could better maintain their viability against apoptosis and had the potential to promote angiogenesis compared with the ECs or SMC spheroids. The 3D migration of cell spheroids in hydrogels offers a unique platform to disclose the relationship between cell-cell and cell-substrate interactions simultaneously and thereby can better mimic the real situation of tissue regeneration *in vivo*.

## 5. Materials and Methods

### 5.1. Materials

Hyaluronic acid (HA, Mw = 100 kDa, Dongyuan Biotechnology Inc., Zhejiang, China), sodium chloride, disodium hydrogen phosphate dodecahydrate, potassium chloride, potassium dihydrogen phosphate, calcium chloride, and N,N-dimethylformamide were purchased from Sinopharm Chemical Reagent Co., Ltd (China). Human fibrinogen was purchased from Jiangxi Boya Biopharmaceutical Co., Ltd. The following materials were used as received: initiator Irgacure 2959 (BASF, Germany), methacrylic anhydride (MA, Aladdin USA, CAS# 760-93-0), thrombin (EMD Millipore Corp, USA), Pluronic® F-127 (Sigma-Aldrich, Germany, CAS# 9003-11-6), Dulbecco's modified Eagle's medium (DMEM, Gibco), penicillin (CSPC PHARMA) and streptomycin (Lukang PHARMA), fetal bovine serum (FBS, Sijiqing Inc., Hangzhou, China), lipopolysaccharide (LPS, Escherichia coli O111:B4, catalog no. L2630), bovine serum albumin (BSA, AMResco, CAS# 9048-46-8), 4′,6-diamidino-2-phenylindole (DAPI, Sigma), rhodamine phalloidin (Invitrogen), and Triton X-100 (Sigma-Aldrich, CAS# 9002-93-1), Cell Tracker™ Green CMFDA (5-chloromethylfluorescein diacetate, Invitrogen by Thermo Fisher Scientific, USA), Cell Tracker™ Orange CMTMR (5-(6)-(((4-chloromethyl) benzoyl) amino) tetramethylrhodamine, Invitrogen by Thermo Fisher Scientific, USA), fluorescein diacetate (FDA, Sigma-Aldrich, USA), propidium iodide (PI, Sigma-Aldrich, USA), and fluorescein isothiocyanate (FITC, Sigma-Aldrich, USA). The water used in the experiments was purified by a Milli-Q water system (Millipore, Darmstadt, Germany).

### 5.2. Fabrication and Characterization of Hydrogels

First, the methacrylated hyaluronic acid (HA-MA) was synthesized via an esterification method, whose structure was characterized in [Supplementary-material supplementary-material-1]. The composite hydrogels were prepared by combining HA-MA with human fibrinogen (Fg). In brief, the HA-MA and Fg were dissolved in PBS at a concentration of 4% (*W*/*V*) and 15% (*W*/*V*), respectively. The photoinitiator I2959 was dissolved in HA-MA solution with a concentration of 0.05% (*W*/*V*). Thrombin was dissolved in 50 mM CaCl_2_ with a concentration of 25 U/mL. Three groups of composite hydrogels with different HA-MA/Fg ratios were prepared: HA-MA/Fg(1/3) (50 *μ*L HA-MA, 75 *μ*L Fg, and 75 *μ*L thrombin solutions), HA-MA/Fg(1/1) (100 *μ*L HA-MA, 50 *μ*L Fg, and 50 *μ*L thrombin solutions), and HA-MA/Fg(3/1) (150 *μ*L HA-MA solution, 25 *μ*L Fg solution, and 25 *μ*L thrombin solutions). The designed volumes of solutions were added into a quartz tube with a diameter of 7.2 mm, which were stirred for 1 min to form a homogeneous mixture solution. After the mixture was maintained in an oven at 37°C for 10 min to crosslink the fibrinogen component by thrombin, it was irradiated by 365 nm light (INTELLI-RAY 400, Uvitron, 30 mW/cm^2^) for 2 min to crosslink the second HA-MA networks, resulting in the composite hydrogels. According to literatures [76] and our own experience, these crosslinking conditions would not influence significantly on the cell viability.

The compressive modulus and strength of the hydrogels were determined by a mechanical tester (Instron 5543A) with a compressive rate of 0.5 mm min^−1^. The compressive modulus was calculated before 5-10% strain [54]. To determine the degradation, the composite hydrogels were lyophilized to obtain their original weight (*W*_0_). They were then incubated in Milli-Q water with refreshment of the water every day, and their weight (*W*) was measured at 1, 5, 9, and 14 d after lyophilization, respectively. The degradation degree was calculated by (*W*_0_‐*W*)/*W*_0_ × 100. The weight of hydrogels was measured as *W*_1_ after wiping off gently the residual liquid around the hydrogels with filter papers, and then the weight of lyophilized hydrogels was measured as *W*_0_. The swelling ratio was calculated by (*W*_1_‐*W*_0_)/*W*_0_. Each value was averaged from three parallel samples. The fibrinogen biomacromolecules were labeled with FITC [77], so that the distribution of obtained fibrin networks could be observed by confocal laser scanning microscopy (CLSM, LSM-510, Zeiss).

### 5.3. Cell Culture and Cell Spheroid Fabrication

Standard cell lines of human vein endothelial cells (ECs) and human vascular smooth muscle cells (SMCs) were obtained from the Cell Bank of Typical Culture Collection of Chinese Academy of Sciences (Shanghai, China). The ECs and SMCs were maintained in high-glucose DMEM supplemented with 10% fetal bovine serum (FBS, Zhejiang Tianhang Biotechnology), 100 *μ*g/mL streptomycin, and 100 U/mL penicillin.

The cell spheroids were fabricated through culturing cells in a special round-bottom 96-well plate (WHB-96-U1). The surface of the well is hydrophobic and is usually used for suspended cell culture [78]. In this study, each well was treated with 200 *μ*L 5% (*W*/*V*) Pluronic® F-127 aqueous solution at 37°C for 24 h to allow the resistance of cell attachment on the well. After extensively washing and UV sterilization, 1.0 × 10^4^ cells (ECs, SMCs, or 5 × 10^3^ ECs and 5 × 10^3^ SMCs) in 200 *μ*L medium were added into each well and cultured for 24 h to allow the formation of one cell spheroid, which contained 1.0 × 10^4^ cells.

### 5.4. Characterization of Cell Spheroids

After the cell spheroids were transferred into a 48-well plate with 5 mL plastic dropper, they were stained with fluorescein diacetate (FDA, 5 *μ*g/mL) and propidium iodide (PI, 5 *μ*g/mL) for 5 min for live/dead assay and size quantification. After washed with PBS for 3 times, the stained cell spheroids were observed under a fluorescence microscope (IX81, Olympus).

The cell spheroids after preparation or after culture for 3 days on TCPS were also observed by CLSM (LSM-510, Zeiss). First, the samples used in CLSM characterization were fixed with 4% paraformaldehyde at 37°C for 30 min and followed by washing with PBS three times. Then, they were permeabilized by 0.5% Triton X-100/PBS for 5-10 min. After washed with PBS, the samples were incubated in 1% BSA/PBS for 1 h at 37°C to block nonspecific interactions. Finally, they were stained with rhodamine-labeled phalloidin (Invitrogen, USA) and 4,6′-diamino-2-phenylindole (DAPI, Sigma-Aldrich, USA) at 4°C for 12 h, followed by 3 washes in PBS.

The cell proliferation ability of the cell spheroids was determined as well. In brief, after the cell spheroids were transferred to a normal 48-well plate (one cell spheroid in one well), they were cultured routinely. The cytoviability was measured by MTT assay at 2, 4, and 7 days [79]. To better discriminate cell proliferation, the DNA contents of the cell spheroids after being cultured for 2, 4, and 7 days were also measured [80].

### 5.5. Encapsulation of Cell Spheroids in Hydrogels In Situ

The cell spheroids were encapsulated in the hydrogels to study the three-dimensional migration behaviors of cells. The as-prepared EC, SMC, and EC-SMC spheroids were transferred into a 48-well plate and kept two cell spheroids in each well. The designed volumes of Fg, HA-MA, and thrombin were added into each well and stirred for 1 min to obtain a homogeneous mixture with suspended cell spheroids. The hydrogels were formed by incubating at 37°C for 10 min, following with irradiation by 365 nm light for 2 min. After being supplemented with culture medium, the 48-well plate was maintained at 37°C and 5% CO_2_ humidified atmosphere to culture the cell spheroids in the HA-MA/Fg(1/3), HA-MA/Fg(1/1), and HA-MA/Fg(3/1) composite hydrogels.

### 5.6. Migration Assay of the Cell Spheroids in Hydrogels

The migration distance of cells from the cell spheroids encapsulated in the composite hydrogels at 4, 7, and 14 days was measured by CLSM. At each desired time point, the cell spheroids in the hydrogels were fixed with 4% paraformaldehyde at 4°C for 12 h, followed by washing with PBS three times. The cells were permeabilized by 0.5% Triton X-100/PBS for 5-10 min. After washed with PBS, the samples were incubated in 1% BSA/PBS for 1 h at 37°C to block nonspecific interactions. Finally, the samples were stained with rhodamine-labeled phalloidin (Invitrogen, USA) and DAPI at 4°C for 12 h, followed by 3 washes in PBS. The migration distance was measured from the CLSM images, which was calculated by the difference of diameters of the cell contour and the original cell spheroids.

### 5.7. Real-Time Quantitative Polymerase Chain Reaction (qRT-PCR) Analysis

The gene expression profiles of *α*-actin, *β*1 integrin, vimentin, MMP-1, CD44, Bcl-XL, SDF-1, HIF-1*α*, and angiotensin 1 were examined by qRT-PCR. Briefly, after the three types of cell spheroids were encapsulated in the HA-MA/Fg(1/1) hydrogels for 7 days, the single-cell sequence-specific amplification kit (Vazyme, China) was used to obtain cDNAs, which were adopted as templates for the subsequent RT-PCR amplifications. The RT-PCR reactions were performed by CFX96 (Bio-Rad, USA) and the SYBR Premix EX TaqTM kit (Takara, China). 18S ribosomal subunit was used as the endogenous reference housekeeping gene. The relative gene expression levels were analyzed by the comparative DDCT (threshold cycle) method and normalized to the housekeeping gene. The primers were designed by Primer Premier 6 software (Premier Biosoft, USA) and tabulated in [Supplementary-material supplementary-material-1].

### 5.8. Statistical Analysis

All experiments were conducted independently three or more times with triplicate samples unless specially mentioned. Results are reported as the mean ± standard deviation. Statistical analysis was performed using one-way analysis of variance (ANOVA) with Tukey's post hoc method. A few comparisons were also made between individual groups with *t*-test. *p* < 0.05 was considered statistically significant.

## Supplementary Materials

Supplementary Materials Table S1: primers used in PCR. Figure S1: (a) synthesis route of HA-MA. (b) 1H NMR spectra of HA and HA-MA. Figure S2: representative stress-strain curves of (a) HA-MA/Fg(1/3), (b) HA-MA/Fg(1/1), and (c) HA-MA/Fg(3/1) composite hydrogels. Figure S3: SEM images of lyophilized (a) HA-MA/Fg(1/3), (b) HA-MA/Fg(1/1), and (c) HA-MA/Fg(3/1) composite hydrogels. Table S2: the area of characteristic peaks. Figure S4b: modulus of composite hydrogels after degradation for 1 day. ∗ indicates significant difference at *p* < 0.05 level. Figure S5: optical images of (a, b) EC, (b, e) SMC, and (c, f) EC-SMC spheroids being cultured on normal culture plates for 4 days. (d–f) Higher magnification images of (a–c), respectively. Figure S6: representative fluorescent images of EC-SMC spheroids being cultured on 48-well plate for 4 days. Green and red represent (a, d) ECs and (b, e) SMCs, respectively. ECs and SMCs were stained by Cell Tracker^TM^ Green CMFDA and Cell Tracker^TM^ Orange CMTMR before they were used to prepare the composite spheroids. (c, f) Merged images of (a) and (b), and (d) and (e), respectively. The images of (a–c) and (d, e) were taken far away and nearby the cell spheroids, respectively. Table S3: cell migration rate at different time periods. Figure S7: migration distance of EC, SMC, and EC-SMC spheroids being cultured on TCPS for different times. ∗ indicates significant difference at *p* < 0.05 level. Figure S8: CLSM images for EC-SMC spheroids in the HA-MA/Fg(1/1) hydrogel with different inhibitors at 7 days after the cytoskeleton was stained with rhodamine-labeled phalloidin (red). Three parallel samples for (a–c) control and addition of (d–f) anti-CD44 and (g–i) free RGD were testified for each group. Figure S9: CLSM images for EC-SMC spheroids in the HA-MA/Fg(1/1) hydrogel with different inhibitors at 7 days after the cytoskeleton was stained with rhodamine-labeled phalloidin (red). Three parallel samples for (a–c) control and addition of (d–f) anti-N-cadherin and (g–i) GM6001 were testified for each group.
