# Supplementary material for: Spheroids of Endothelial Cells and Vascular Smooth Muscle Cells Promote Cell Migration in Hyaluronic Acid and Fibrinogen Composite Hydrogels
Source: Research (Wash D C). 2020 Feb 19;2020:8970480. doi: 10.34133/2020/8970480 (PMC7049785; doi:10.34133/2020/8970480)
Supplement: Supplementary Materials — Table S1: primers used in PCR. Figure S1: (a) synthesis route of HA-MA. (b) 1H NMR spectra of HA and HA-MA. Figure S2: representative stress-strain curves of (a) HA-MA/Fg(1/3), (b) HA-MA/Fg(1/1), and (c) HA-MA/Fg(3/1) composite hydrogels. Figure S3: SEM images of lyophilized (a) HA-MA/Fg(1/3), (b) HA-MA/Fg(1/1), and (c) HA-MA/Fg(3/1) composite hydrogels. Table S2: the area of characteristic peaks. Figure S4b: modulus of composite hydrogels after degradation for 1 day. ∗ indicates significant difference at p < 0.05 level. Figure S5: optical images of (a, b) EC, (b, e) SMC, and (c, f) EC-SMC spheroids being cultured on normal culture plates for 4 days. (d–f) Higher magnification images of (a–c), respectively. Figure S6: representative fluorescent images of EC-SMC spheroids being cultured on 48-well plate for 4 days. Green and red represent (a, d) ECs and (b, e) SMCs, respectively. ECs and SMCs were stained by Cell TrackerTM Green CMFDA and Cell TrackerTM Orange CMTMR before they were used to prepare the composite spheroids. (c, f) Merged images of (a) and (b), and (d) and (e), respectively. The images of (a–c) and (d, e) were taken far away and nearby the cell spheroids, respectively. Table S3: cell migration rate at different time periods. Figure S7: migration distance of EC, SMC, and EC-SMC spheroids being cultured on TCPS for different times. ∗ indicates significant difference at p < 0.05 level. Figure S8: CLSM images for EC-SMC spheroids in the HA-MA/Fg(1/1) hydrogel with different inhibitors at 7 days after the cytoskeleton was stained with rhodamine-labeled phalloidin (red). Three parallel samples for (a–c) control and addition of (d–f) anti-CD44 and (g–i) free RGD were testified for each group. Figure S9: CLSM images for EC-SMC spheroids in the HA-MA/Fg(1/1) hydrogel with different inhibitors at 7 days after the cytoskeleton was stained with rhodamine-labeled phalloidin (red). Three parallel samples for (a–c) control and addition of (d–f) anti-N-cadherin and (g [file 8970480.f1.doc]

**Supporting information**

**Spheroids of endothelial cells and vascular smooth muscle cells promote 3D cell migration in hyaluronic acid and fibrinogen composite hydrogels**

Xingang Zuo^1^, Haolan Zhang^1^, Yiyuan Duan^1,2^, Hao Shou^1^, Shan Yu^1^, Changyou Gao^1,2^*

^1^ MOE Key Laboratory of Macromolecular Synthesis and Functionalization, Department of Polymer Science and Engineering, Zhejiang University, Hangzhou 310027, China

^2^ Dr. Li Dak Sum & Yip Yio Chin Center for Stem Cell and Regenerative Medicine, Zhejiang University, Hangzhou 310058, China

E-mail: [cygao@zju.edu.cn](mailto:cygao@zju.edu.cn)

**Table S1** Primers used in PCR

| **Gene** | **Primer sequences (forward and reverse)** |
| --- | --- |
| Bcl-2 | 5'(FAM)-TGT GCG CGC GTA TAA ATT GCC GA-(TAMRA)p3'  AAG CGG TCC CGT GGA TAG A  TCC GGT ATT CGC AGA AGT CC |
| Bcl-XL | 5'(FAM)-TGC GTG GAA AGC GTA GAC AAG GAG ATG C-(TAMRA)p3'  GAG GCA GGC GAC GAG TTT GAA  GGG GTG GGA GGG TAG AGT GGA |
| SDF-1 | GTG TCA CTG GCG ACA CGT AG  TCC CAT CCC ACA GAG AGA AG |
| HIF-1α | CCA TTA GAA AGC AGT TCC GC  TGG GTA GGA GAT GGA GAT GC |
| Ang1 | GGG GGA GGY YGG ACT GTA AT  AGG GCA CAT TTG CAC ATA CA |
| MMP-1 | TGC TCA TGC TTT TCA ACC AG  TCC ACT TCT GGG TAC AAG GG |
| Integrin β1 | CCTACTTCTGCACGATGTGATG  CCTTTGCTACGGTTGGTTACATT |
| CD44 | TACAGCATCTCTCGGACGGA  CACCCCTGTGTTGTTTGCTG |
| vimentin | TGGACCAGCTAACCAACGAC  GCCAGAGACGCATTGTCAAC |
| α-actin | GTGTTGCCCCTGAAGAGCAT  GCTGGGACATTGAAAGTCTCA |
| 18S | ATCACCATTATGCAGAATCCACG  GACCTGGCTGTATTTTCCATCC |

**Figure S1** (a) Synthesis route of HA-MA. (b) ^1^H NMR spectra of HA and HA-MA.

The methacrylated hyaluronic acid (HA-MA) was synthesized by reacting hyaluronic acid (HA) with methacrylic anhydride according to a previous report with slight modification[1]. Briefly, 1.0 g HA was added into a 250 mL three-necked flask with the mixture of 60 mL MilliQ water and 30 mL DMF, and thoroughly dissolved under agitation. The flask was placed into an ice/water bath with continuous agitation, into which 8 mL MA was added in dropwise. The pH value of the solution was maintained in the range of 8-9 by adding 5M NaOH during the whole process of reaction. The reaction was maintained for 24 h after the MA was added completely. The solution was then poured into ice ethanol to obtain the MA-HA precipitate, which was collected via centrifugation at 8000 rpm. The MA-HA was dialyzed in a tube with a cut-off molecular weight of 3.5 kDa against water for 7 days before lyophilization. The molecular structure was characterized by ^1^H NMR (Figure S1b) (Burker DMX -500, solvent is DO_2_), according to which the percentage of double bond in repeat unit of HA was found to be about 50%.

**Figure S2** Representative stress-strain curves of (a) HA-MA/Fg(1/3), (b) HA-MA/Fg(1/1) and (c) HA-MA/Fg(3/1) composite hydrogels.

**Figure S3** SEM images of lyophilized (a) HA-MA/Fg(1/3), (b) HA-MA/Fg(1/1) and (c) HA-MA/Fg(3/1) composite hydrogels.

**Figure S4a** FTIR spectra of composite hydrogels before (HA-MA/Fg(1/3), HA-MA/Fg(1/1) and HA-MA/Fg(3/1)) and after degradation (D-HA-MA/Fg(1/3), D-HA-MA/Fg(1/1) and D-HA-MA/Fg(3/1)) for 1 day.

Fourier transfer infrared (FTIR) spectroscopy (Bruker Tensor 27, Switzerland) was used to characterize the variation of hydrogels before and after degradation. The wide peak over 3000 cm^-1^ is assigned to the stretching vibration of N-H and O-H in MA-HA and Fg. The peak at 1660 cm^-1^ is stretching vibration of C=O of MA and fibrinogen. The peak at 1167 cm^-1^ is the scissoring vibration of –C=C–H, which is attributed to the grafted methacrylate residues[2].

**Table S2** The area of characteristic peaks

|  | C=O | C=C－H | (C=C－H)/(C=O)**%** |
| --- | --- | --- | --- |
| HA-MA/Fg(1/3) | -92.26709 | -2.32998 | 2.53 |
| D-HA-MA/Fg(1/3) | -82.4745 | -3.60211 | 4.37 |
| HA-MA/Fg(1/1) | -111.95706 | -7.11307 | 6.35 |
| D-HA-MA/Fg(1/1) | -76.07424 | -5.4525 | 7.17 |
| HA-MA/Fg(3/1) | -138.33667 | -12.77891 | 9.24 |
| D-HA-MA/Fg(3/1) | -102.85611 | -10.96637 | 10.66 |

**Figrue S4b** Modulus of composite hydrogels after degradation for 1 day. * indicates significant difference at p < 0.05 level.

**Figure S5** Optical images of (a,b) ECs, (b,e) SMCs, and (c,f) ECs-SMCs spheroids being cultured on normal culture plates for 4 days. (d-f) are higher magnification images of (a-c), respectively.

**Figure S6** Representative fluorescent images of ECs-SMCs spheroids being cultured on 48-well plate for 4 days. Green and red represent (a,d) ECs and (b,e) SMCs, respectively. ECs and SMCs were stained by Cell Tracker^TM^ Green CMFDA and Cell Tracker^TM^ Orange CMTMR before they were used to prepare the composite spheroids. (c) and (f) are merged images of (a) and (b), and (d) and (e), respectively. The images of (a-c) and (d-e) were taken far away and nearby the cell spheroids, respectively.

**Table S3** Cell migration rate at different time periods.

|  |  | 0-4 days  (μm/24h) | 4-7 days  (μm/24h) | 7-14 days  (μm/24h) | 0-14 days  (μm/24h) |
| --- | --- | --- | --- | --- | --- |
| ECs  spheroids | HA-MA/Fg(1/3) | 71.3 | 56.2 | 171.2 | 118.0 |
|  | HA-MA/Fg(1/1) | 49.0 | 430.3 | 6.2 | 106.2 |
|  | HA-MA/Fg(3/1) | 12.0 | 126.9 | 100.5 | 80.9 |
| SMCs  spheroids | HA-MA/Fg(1/3) | 43.5 | 17.7 | 6.7 | 19.6 |
|  | HA-MA/Fg(1/1) | 60.9 | 6.2 | 11.6 | 24.5 |
|  | HA-MA/Fg(3/1) | 46.6 | 24.4 | 20.0 | 28.6 |
| ECs-SMCs  spheroids | HA-MA/Fg(1/3) | 161.0 | 32.8 | 215.7 | 160.9 |
|  | HA-MA/Fg(1/1) | 321.4 | 236.2 | 181.6 | 233.3 |
|  | HA-MA/Fg(3/1) | 84.2 | 77.0 | 244.0 | 162.5 |

**Figure S7** Migration distance of ECs, SMCs and ECs-SMCs spheroids being cultured on TCPS for different time. * indicates significant difference at p < 0.05 level.

**Figure S8** CLSM images for ECs-SMCs spheroids in the HA-MA/Fg(1/1) hydrogel with different inhibitors at 7 days after the cytoskeleton was stained with rhodamine-labeled phalloidin (red). Three parallel samples for (a-c) control, and addition of (d-f) anti-CD44 and (g-i) free RGD were testified for each group.

**Figure S9** CLSM images for ECs-SMCs spheroids in the HA-MA/Fg(1/1) hydrogel with different inhibitors at 7 days after the cytoskeleton was stained with rhodamine-labeled phalloidin (red). Three parallel samples for (a-c) control, and addition of (d-f) anti N-cadherin and (g-i) GM6001 were testified for each group.

**References:**

[1] C.C. Yang, X. Wang, X.K. Yao, Y.J. Zhang, W. Wu, X.Q. Jiang et al., "Hyaluronic acid nanogels with enzyme-sensitive cross-linking group for drug delivery", *Journal of Controlled Release,* vol. 205, pp. 206-217, 2015.

[2] A. Morelli, F. Chiellini et al., "Ulvan as a New Type of Biomaterial from Renewable Resources: Functionalization and Hydrogel Preparation," *Macromolecular Chemistry and Physics,* vol. 211, no. 7, pp. 821-832, 2010.
